# Plastome organization and evolution of chloroplast genes in *Cardamine* species adapted to contrasting habitats

**DOI:** 10.1186/s12864-015-1498-0

**Published:** 2015-04-17

**Authors:** Shiliang Hu, Gaurav Sablok, Bo Wang, Dong Qu, Enrico Barbaro, Roberto Viola, Mingai Li, Claudio Varotto

**Affiliations:** Ecogenomics Laboratory, Department of Biodiversity and Molecular Ecology, Research and Innovation Centre, Fondazione Edmund Mach, Via E. Mach 1, 38010 S Michele all’Adige (TN), Italy; College of Horticulture, Northwest Agricultural and Forest University, 712100 Yangling, Shaanxi PR China

**Keywords:** *Cardamine*, Molecular adaptation, Large single copy region (LSC), Small single copy region (SSC), Plastomes, Positive selection, Repeats, Codon usage

## Abstract

**Background:**

Plastid genomes, also known as plastomes, are shaped by the selective forces acting on the fundamental cellular functions they code for and thus they are expected to preserve signatures of the adaptive path undertaken by different plant species during evolution. To identify molecular signatures of positive selection associated to adaptation to contrasting ecological niches, we sequenced with Solexa technology the plastomes of two congeneric Brassicaceae species with different habitat preference, *Cardamine resedifolia* and *Cardamine impatiens*.

**Results:**

Following in-depth characterization of plastome organization, repeat patterns and gene space, the comparison of the newly sequenced plastomes between each other and with 15 fully sequenced Brassicaceae plastomes publically available in GenBank uncovered dynamic variation of the IR boundaries in the *Cardamine* lineage. We further detected signatures of positive selection in ten of the 75 protein-coding genes of the examined plastomes, identifying a range of chloroplast functions putatively involved in adaptive processes within the family. For instance, the three residues found to be under positive selection in RUBISCO could possibly be involved in the modulation of RUBISCO aggregation/activation and enzymatic specificty in Brassicaceae. In addition, our results points to differential evolutionary rates in *Cardamin*e plastomes.

**Conclusions:**

Overall our results support the existence of wider signatures of positive selection in the plastome of *C. resedifolia*, possibly as a consequence of adaptation to high altitude environments. We further provide a first characterization of the selective patterns shaping the Brassicaceae plastomes, which could help elucidate the driving forces underlying adaptation and evolution in this important plant family.

**Electronic supplementary material:**

The online version of this article (doi:10.1186/s12864-015-1498-0) contains supplementary material, which is available to authorized users.

## Background

Chloroplast genomes, hereafter referred to as plastomes, have been widely used as models for elucidating the patterns of genetic variation in space and time, ranging from colonization to speciation and phylogeny, encompassing both micro- and macro-evolutionary events across all lineages of plants [1]. Understanding the phyletic patterns of chloroplast evolution can also potentially layout the basis of species discrimination [2], as indicated by the fact that the core DNA barcode chosen for plants is composed by the two plastomic regions *rbc*L and *mat*K [3]. In fact, the presence of a high number of plastomes per cell, ease of amplification across the angiosperm phylogeny, and good content in terms of phylogenetic information explain the popularity of these and other plastidial markers for both species identification and phylogenetic reconstruction. The organization of the plastome is remarkably conserved in higher plants, and it is characterized by two usually large inverted repeat regions (IR_A_ and IR_B_) separated by single copy regions of different lengths, called large single copy region (LSC) and small single copy region (SSC; [4]). Both traditional Sanger sequencing and next generation sequencing approaches have been widely employed to elucidate the dynamic changes of these four plastome regions, revealing patterns of evolutionary expansion and contraction in different plant lineages [5,6]. The genes present in plastomes play fundamental functions for the organisms bearing them: they encode the core proteins of photosynthetic complexes, including Photosystem I, Photosystem II, Cytochrome b_6_f, NADH dehydrogenase, ATP synthase and the large subunit of *RUBISCO*, tRNAs and ribosomal RNAs and proteins necessary for chloroplast ribosomal assembly and translation, and sigma factors necessary for transcription of chloroplast genes [7]. Plastomes of seed plants typically encode four rRNAs, around 30 tRNAs and up to 80 unique protein-coding genes [6-8]. With the notable exception of extensive photosynthetic gene loss in parasitic plants [9], genic regions are generally conserved across the plastomes of higher plants reported so far; inversions and other rearrangements, however, are frequently reported [5]. In line with the higher conservation of genic versus inter-genic regions, a recent report of plastome from basal asterids indicates the conservation of the repeat patterns in the coding regions, whereas the evolution of the repeats in the non-coding regions is lineage-specific [10]. Due to the endosymbiotic origin of plastomes, several of the genes are coordinately transcribed in operons (e.g. the *psb*B operon) [11,12]. Additionally, chloroplast transcripts undergo RNA editing, especially in ancient plant lineages like ferns and hornworts [13,14].

The *Cardamine* genus represents one of the largest and most polyploid-rich genera of the Brassicaceae, and underwent several recent and rapid speciation events contributing to the divergent evolution of its species [15]. The diversification of *Cardamine* has been driven by multiple events of polyploidization and hybridization, which, together with the high number of species, has till now hindered the obtainment of a comprehensive phylogeny of the genus [16]. Using cpDNA regions, patterns of extensive genetic variation have been previously reported in *Cardamine flexuosa* and related species [17]. The high seed production characterizing several *Cardamine* taxa makes them highly invasive species, which can become noxious in both wild habitats and cultivation. *C. flexuosa* and *C. hirsuta*, for instance, are among the most common weeds in cultivation [17]. *C. impatiens* is rapidly colonizing North America, where it is considered as one of the most aggressive invaders of the understory given its high adaptability to low light conditions [18]. Several *Cardamine* species have been object of growing interest as models for evolutionary adaptive traits and morphological development. *C. hirsuta*, a cosmopolitan weed with fast life cycle, is now a well established model for development of leaf dissection in plants [19]. *C. flexuosa* has been recently used to elucidate the interplay between age and vernalization in regulating flowering [20]. Earlier, in a pioneering study with cross-species microarray hybridization, the whole transcriptome of *C. kokaiensis* provided insights on the molecular bases of cleistogamy and its relationship with environmental conditions, especially chilling temperatures [21].

More recently, using the *Cardamine* genus as a model we demonstrated transcriptome-wide patterns of molecular evolution in genes pertaining to different environmental habitat adaptation by comparative analysis of low altitude, short lived, nemoral species *C. impatiens* to high altitude, perennial, open-habitat dweller *C. resedifolia*, suggesting contrasting patterns of molecular evolution in photosynthetic and cold-tolerance genes [22]. The results explicitly demonstrated faster evolution of the cold-related genes exclusively in the high altitude species *C. resedifolia* [22]. To extend the understanding of positive selection signatures observed in the aforementioned transcriptome-wide analysis to organelles, in this study we carried out the complete sequencing with Solexa technology of the plastome of both species and characterized their gene space and repeat patterns. The comparison of the newly sequenced plastomes between each other and with 15 fully sequenced Brassicaceae plastomes publically available in GenBank uncovered dynamic variation of the IR boundaries in the *Cardamine* lineage associated to generation of lineage-specific pseudogenic fragments in this region. In addition, we could detect signatures of positive selection in ten of the 75 protein-coding genes of the plastomes examined as well as specific *rbcL* residues undergoing intra-peptide co-evolution. Overall our results support the existence of wider signatures of positive selection in the plastome of *C. resedifolia*, possibly as a consequence of adaptation to high altitude environments.

## Results and discussion

### Genome assembly and validation

In order to further our understanding of selective patterns associated to contrasting environmental adaptation in plants, we obtained and annotated the complete plastome sequence of two congeneric species, high altitude *Cardamine resedifolia* (GenBank accession number KJ136821) and low altitude *C. impatiens* (accession number KJ136822). The primers used amplified an average of 6,2 Kbp, with a minimum and maximum amplicon length of 3,5 and 9,0 Kbp, respectively (Additional file 1: Table S1). In this way, a total of 650335 x100 bp paired-end (PE) reads with a Q30 quality value and mean insert size of 315 bp were obtained for *C. resedifolia*, while 847076 x100 bp PE reads with 325 bp insert size were obtained for *C. impatiens.* Velvet *de-novo* assembly resulted in 36 and 48 scaffolds in *C. resedifolia* and *C. impatiens*, respectively (Table 1). To validate the accuracy of the assembled plastome we carried out Sanger sequencing of PCR amplicons spanning the junction regions (LSC/IR_A_, LSC/IR_B_, SSC/ IR_A_, SSC/IR_B_). The perfect identity of the sequences to those resulting from assembly confirmed the reliability of assembled plastomes (data not shown). Additionally, we Sanger-sequenced selected regions of the plastome genic space to verify the correct translational frame of the coding regions and to eliminate any Ns still present in the assembly. The finished, high quality organelle genome sequences thus obtained were used for downstream analyses.

**Table 1:** **Sequencing statistics and general characteristics of** ***C. resedifolia*** **and** ***C. impatiens*** **plastome assembly**

	***C. resedifolia***	***C. impatiens***
PE reads with a Q > 30	650335 (315 bp*)	847076 (325 bp*)
Type of Assembler	de-bruijn Graph	de-bruijn Graph
*K-mer* used	63	63
Number of scaffolds	36	48
Reference species	*Nasturtium officinale*	*Nasturtium officinale*
Assembled plastome size	155036 bp	155611 bp
Number of genes	85(79unique)	85(79unique)
Number of t-RNA	37(30unique)	37(30unique)
Number of r-RNA	8(4unique)	8(4unique)
Length of IRa and IRb	26502 bp	26476 bp
Length of SSC	17867 bp	17948 bp
Length of LSC	84165 bp	84711 bp
Annotation	cpGAVAS, DOGMA	CpGAVAS, DOGMA

*Number in parenthesis indicate the insert size of the PE library.

### Plastome structural features and gene content

**Figure 1:**
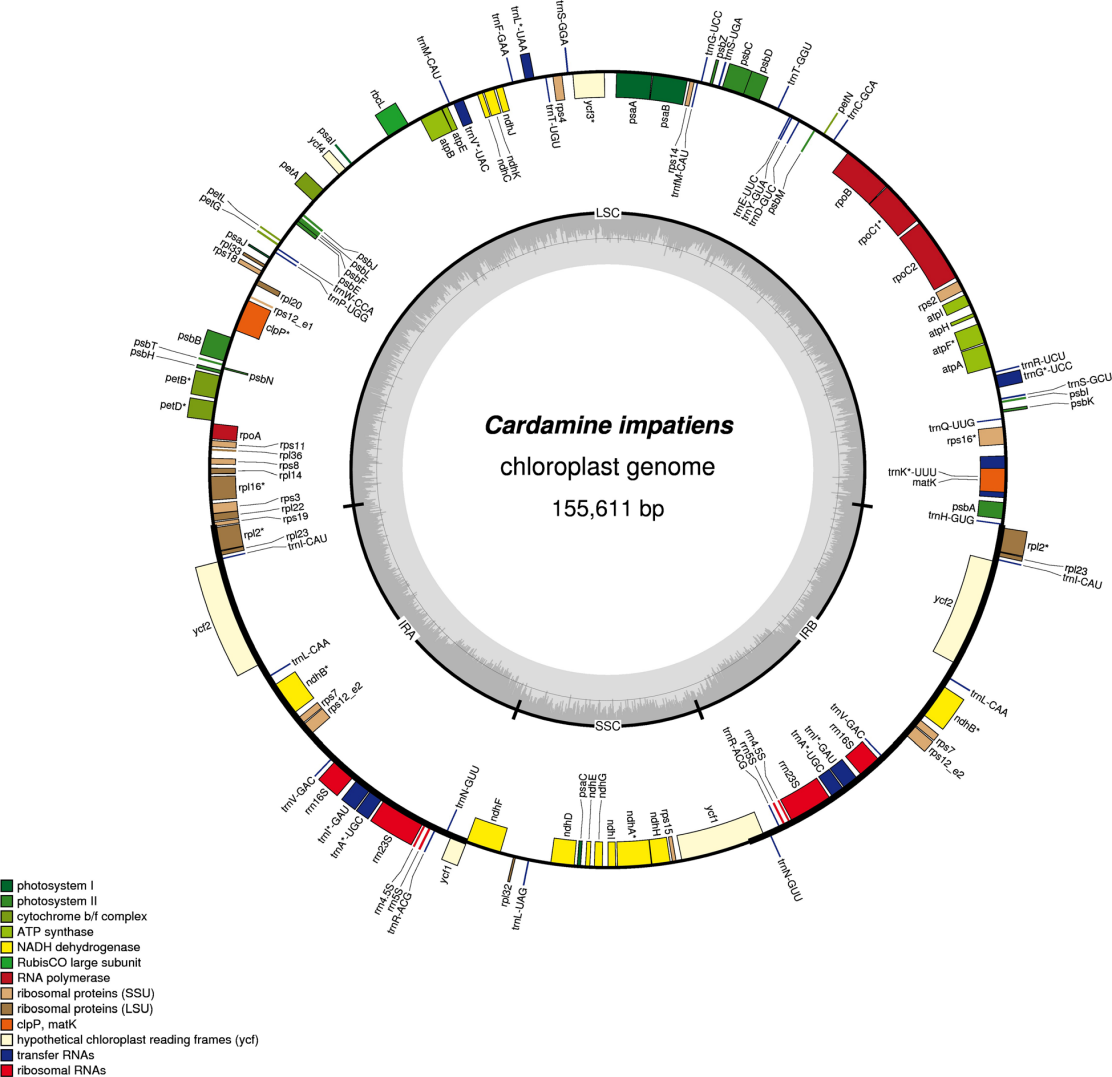
Plastome map of *C. resedifolia*. Genes shown outside of the larger circle are transcribed clockwise, while genes shown inside are transcribed counterclockwise. Thick lines of the smaller circle indicate IRs and the inner circle represents the GC variation across the genic regions.

**Figure 2:**
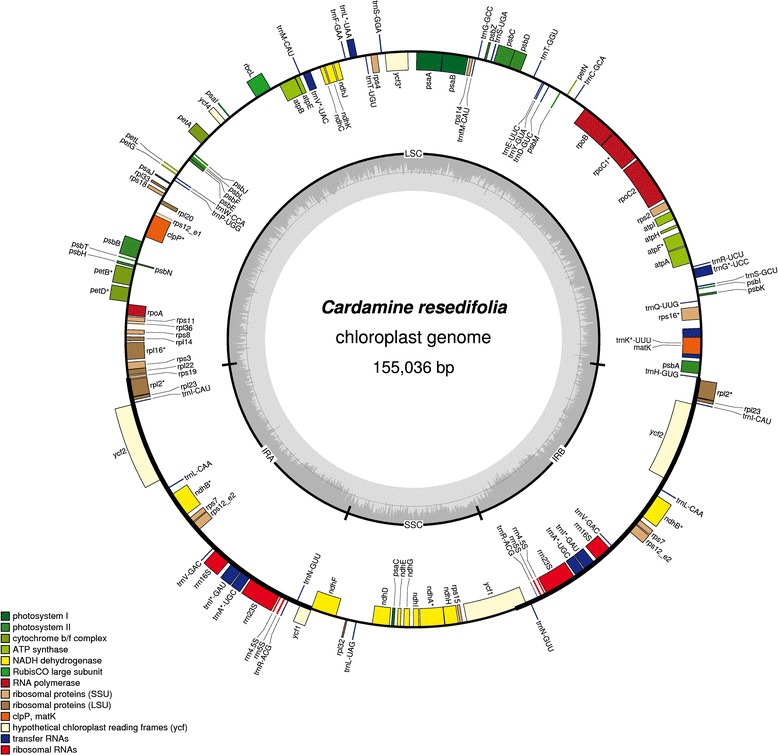
Plastomic map of *C. impatiens*. Genes shown outside of the larger circle are transcribed clockwise, while genes shown inside are transcribed counterclockwise. Thick lines of the smaller circle indicate IRs and the inner circle represents the GC variation across the genic regions.

The finished plastomes of *C. resedifolia* and *C. impatiens* have a total length of 155036 bp and 155611 bp and a GC content of 36.30% and 36.33%, respectively. These values of GC content suggest an AT-rich plastome organization, which is similar to the other Brassicaceae plastomes sequenced so far (Figures 1 and 2). Quadripartite organization of plastomes, characterized by two large inverted repeats, plays a major role in the recombination and the structural diversity by gene expansion and gene loss in chloroplast genomes [8]. Each plastome assembly displayed a pair of inverted repeats (IR_A_ and IR_B_) of 26502 bp and 26476 bp respectively in *C. resedifolia* and *C. impatiens*, demarking large single copy (LSC) regions of 84165 bp and 84711 bp and small single copy (SSC) regions of 17867 bp and 17948 bp in *C. resedifolia* and *C. impatiens* respectively (Table 1, Additional file 2: Table S2). The assembled plastomes contained a total of 85 protein-coding genes, 37 t-RNAs, and 8 r-RNAs in both *C. resedifolia* and *C. impatiens*. We observed a total of 12 protein-coding regions and 6 t-RNAs containing one or more introns (Table 2), which is similar to *Nicotiana tabacum*, *Panax ginseng* and *Salvia miltiorrhiza* [23] but higher than the basal plastomes of the Asterid lineage, where only *ycf*3 and *clpP* have been reported to be protein-coding genes with introns [10]. Of the observed gene space in *C. resedifolia* and *C. impatiens*, 79 protein-coding genes, 30 t-RNA and 4 r-RNAs were found to be unique while 6 protein-coding (*ndhB*, *rpl23*, *rps7*, *rps12*, *ycf2*, *rpl2*), 7 t-RNAs (*trnA-UGC, trnI-CAU, trnI-GAU, trnL-CAA, trnN-GUU, trnR-ACG and trnV-GAC*) and 4 r-RNA genes (*rrn4.5, rrn5, rrn16, rrn23*) were found be duplicated in IR_A_ and IR_B_ (Table 2). GC content analysis of the IR, SSC and LSC showed no major fluctuations, with SSC regions accounting for 29.26%/29.16% GC, LSC 34.06%/34.00%, IR_A_ and IR_B_ each accounting for 42.36%/42.36% GC in *C. impatiens* and *C. resedifolia*, respectively. Of the observed intron-containing genes, *clpP* and *ycf3* contained two introns. In *rps12* a trans-splicing event was observed with the 5′ end located in the LSC region and the duplicated 3′ end in the IR region as previously reported in *Nicotiana* [24]. In the *trnK-UUU* gene was located the largest intron, harboring the *mat*K gene and accounting for 2552 bp in *C. resedifolia* and 2561 bp in *C. impatiens* (Additional file 3: Table S3).

**Table 2:** **List of genes encoded in** ***C. impatiens*** **and** ***C. resedifolia*** **plastomes**

**Gene Category**	**Genes**
ribosomal RNAS	^*§*^ *rrn4.5,* ^*§*^ *rrn5,* ^*§*^ *rrn16,* ^*§*^ *rrn23*
transfer RNAs	^*§*^ *trnA-UGC, trnC-GCA, trnD-GUC, trnE-UUC, trnF-GAA, trnfM-CAU, *trnG-UCC, trnG-UCC, trnH-GUG, ^*§*^ *trnI-CAU,* ^*§*^ *trnI-GAU, *trnK-UUU, ^*§*^ trnL-CAA, *trnL-UAA, trnL-UAG, trnM-CAU, ^*§*^ *trnN-GUU, trnP-UGG, trnQ-UUG,* ^*§*^ trnR-ACG, trnR-UCU, trnS-GCU, trnS-UGA, trnS-GGA, trnT-UGU, trnT-GGU, *trnV-UAC, ^*§*^ *trnV-GAC, trnW-CCA, trnY-GUA*
Photosystem I	*psaA, psaB, psaC, psaI, psaJ*
Photosystem II	*psbA, psbB, psbC, psbD, psbE, psbF, psbH, psbI, psbJ, psbK, psbL, psbM, psbN, psbT, psbZ*
Cytochrome	petA, *petB, *petD, petG, petL, petN
ATP synthase	atpA, atpB, atpE, *atpF, atpH, atpI
Rubisco	*rbcL*
NADH dehydrogenase	*ndhA, ^*§*^ *ndhB, ndhC, ndhD, ndhE, ndhF, ndhG, ndhH, ndhI, ndhJ, ndhK
Ribosomal protein (large subunit)	^*§*^ *rpl2, rpl14, *rpl16, rpl20, rpl22, ^*§*^ *rpl23, rpl32, rpl33, rpl36*
Ribosomal protein (small subunit)	*rps2, rps3, rps4,* ^*§*^ *rps7, rps8, rps11,* ^*§*^ *rps12, rps14, rps15, *rps16, rps18, rps19
RNA polymerase	rpoA, rpoB, *rpoC1, rpoC2
ATP-dependent protease	*clpP
Cytochrome c biogenesis	*ccsA*
Membrane protein	*cemA*
Maturase	*matK*
Conserved reading frames	*ycf1_short, ycf1_long,* ^*§*^ ycf2, *ycf3, ycf4

^§^Gene completely duplicated in the inverted repeat. *Gene with intron(s).

Pseudogenization events (gene duplication followed by loss of function) have been reported in several plant lineages, e.g., in the plastomes of Anthemideae tribe within the Asteraceae family and *Cocus nucifera*, which belongs to the Arecaceae family [8,25]. Among the genes that underwent pseudogenization there are *ycf68*, *ycf1* and *rps19*, which showed incomplete duplication in the IR_A_/IR_B_ and LSC junction regions with loss of function due to accumulation of premature stop codons or truncations. In both *Cardamine* species a partial duplication (106 bp) of the full-length copy of the *rps19* gene (279 bp) located at the IR_A_/LSC boundary is found in the IR_B_/LSC region. The fact that only one gene copy is present in the outgroup *N. officinale* indicates that the duplication event leading to *rps19* pseudogenization occurred after the split between *Nasturtium* and *Cardamine*. Sequencing of IR_B_/LSC regions from additional *Cardamine* species and closely related outgroups will be required to ascertain whether the psedogenization event is genus-specific or not. The conservation of pseudogene length and the close phylogenetic proximity of *Nasturtium* to *Cardamine* [26], however, point to a relatively recent origin of the causal duplication. The basal position of the clade comprising *C. resedifolia* further corroborates the view that the duplication possibly happened early during the radiation of the *Cardamine* genus [15].

Among the coding regions of the sequenced plastomes, the majority of genes have canonical ATG as *bona-fide* start codons. Only 3 genes (*ndhD*, *psbC*, *rps19*) had non-canonical or conflicting starting codon annotations compared to those in the reference plastomes deposited in GenBank, thus requiring manual curation. Previously, RNA editing events of the AUG initiation site to GUG have been reported for *psbC* [27] and *rps19* [8,25]. Analogously (but not observed in our study), RNA editing events contributing to the change of the translational initiation codon to GUG have been reported also in *cemA* [28]. Previous studies on non-canonical translational mechanisms suggest that translational efficiency of GUG codons is relatively high as compared to canonical AUG as initiation codon [29]. It is, therefore, possible that the GTG start codons observed in Brassicaceae *psbC* and *rps19* are required to ensure enhanced translational efficiency for these genes. Also in the case of *ndhD* we identified a *bona fide* non-canonical start codon (ACG), analogously to what observed in other dicotyledonous and monocotyledonous species [8,30,31]. The reported lack of conservation among congeneric *Nicotiana* species [32] and the ability of unedited *ndhD* mRNA to associate to polysomes [33], however, renders the adaptive relevance of this non-canonical start codon in Brassicaceae elusive.

We further analyzed the codon usage frequency and the relative synonymous codon usage frequency (RSCU) in the two *Cardamine* plastomes. Mutational bias has been reported as an important force shaping codon usage in both animal and plant nuclear genomes [34,35]. Only few studies addressed the role of mutational bias in plant organelles, and earlier evidence pointed to a comparativley larger effect of natural selection in organellar biased usage of codons [36-38]. More recent studies, however, challenge this view and convincingly show that mutational bias can also be a dominant force in shaping the coding capacity of plant organelles and especially of Poaceace plastomes [39,40]. We, therefore, evaluated Nc plots to estimate the role of mutational bias in shaping the codon usage frequency in *C. resedifolia* and *C. impatiens* and found that most of the genes falls below the expected line of Nc, suggesting a relevant role of mutational bias in *C. resedifolia* and *C. impatiens* (Additional file 4: Figure S1). To provide support for the observed mutational bias, statistical analysis invoking Spearman-rank correlations (ρ) were further implemented between Nc and GC_3s_ and were found to be significant in case of *C. resedifolia* (ρ = 0.557, p < 0.01) and *C. impatiens* (ρ = 0.595, p < 0.01). We also evaluated (ρ) between Nc and G_3s_ and positive correlations (ρ = 0.620; *C. impatiens*, ρ = 0.597, *C. resedifolia*) were observed, which demonstrates the role of mutational bias in the biased codon usage frequency in *C. resedifolia* and *C. impatiens*. Taken together, these results indicate that in the two *Cardamine* plastomes sequenced in this study a major role is played by mutational bias, analogously to what suggested in the case of the *Coffea arabica* plastome [41]. Currently we do not have any data on translational efficiency in *Cardamine*, but we cannot exclude it as a possible factor contributing to codon bias in their plastomes as previously suggested in the case of *O. sativa* [42]. Our data, on the other hand, indicate a small fraction of positively selected amino acids (see below), suggesting only marginal contributions of natural selection to codon usage bias in *Cardamine*.

### Distribution of repeat content and SSRs analysis

In addition to the larger repeats constituted by IR_A_ and IR_B_, plastid genomes encompass a number of other repeated sequences. We employed REPUTER for the identification of the repeats, which are > 30 bp using a Hamming distance of 90. A total of 49 and 43 repeats were classified in the *C. impatiens* and the *C. resedifolia* plastome (Additional file 5: Table S4), values which are intermediate between those in Poaceae and Arecaceae and the one in Orchidaceae [8]. Among the perfect repeats, we detected four forward repeats, which are located in the LSC (spacer between *trnL* and *trnF*), and two palindromic repeats also localized in the LSC (spacer between *psbT* and *psbN*; Additional file 5: Table S4). Among the imperfect repeats, we annotated a total of 29 forward tandem repeats with a prevalence of them in the spacer between *trnL* and *trnF* and additional 14 palindromic repeats distributed throughout the plastome of *C. impatiens.* In *C. resedifolia*, we observed only two perfect repeats, both palindromic, located in the LSC (spacer between *petN* and *psbM* and spacer between *psbE* and *petL*; Additional file 5: Table S4). All others were imperfect repeats: 15 forward, two reverse and one compound tandem repeats. Interestingly, in *C. resedifolia* we did not observe the large number of repeats found in the *trnL*/*trnF* spacer of *C. impatiens*. As repeat organization and expansion in plastomes may induce recombination and rearrangements (e.g. in Poaceae and Geraniaceae) [8], the *trnL*/*trnF* spacer appears to be a particularly interesting region to reconstruct micro- and macro-evolutionary patterns in *C. impatiens* and closely related species like *C. pectinata* [43].

We further analyzed the distribution of the simple sequence repeats (SSRs), repetitive stretches of 1-6 bp distributed across nuclear and cytoplasmatic genomes, which are prone to mutational errors in replication. Previously, SSRs have been described as a major tool to unravel genome polymorphism across species and for the identification of new species on the basis of the repeat length polymorphism [44]. Since SSRs are prone to slip-strand mispairing, which is demonstrated as a primary source of microsatellite mutational expansion [45], we applied a length threshold greater than 10 bp for mono-, 4 bp for di- and tri- and 3 minimum repetitive units for tetra-, penta- and hexa-nucleotide repeats patterns. We observed a total of 169 SSRs in *C. resedifolia* and 145 SSRs stretches in *C. impatiens* (Additional file 6: Table S5). The observed number of repetitive stretches is in line with the previous results obtained in Brassicaceae [44,46] and other plastomes [23]. Among the observed repeats, the most abundant pattern was found to be stretches of mononucleotides (A/T) accounting for a total of 81 and 61 stretches of polyadenine (polyA) or polythymine (polyT) (A/T) followed by di-nucleotide patterns accounting for a total of 77 and 71 repetitive units in *C. resedifolia* and *C. impatiens*. Interestingly, we observed a higher tendency of longer repeats to occur species-specifically (see e.g. motifs such as AATAG/ATTCT in *C. resedifolia* and AACTAT/AGTTAT in *C. impatiens*; Additional file 6: Table S5), a possible consequence of their rarity [44,46]. Based on the identified SSR stretches, we provide a total of 127 and 114 SSR primer pairs in *C. resedifolia* and in *C. impatiens*, respectively (Additional file 6: Table S5), which can be used for future in-depth studies of phylogeography and population structure in these species.

#### Synteny conservation and phylogeny of sequenced Brassicaceae plastomes

**Figure 3:**
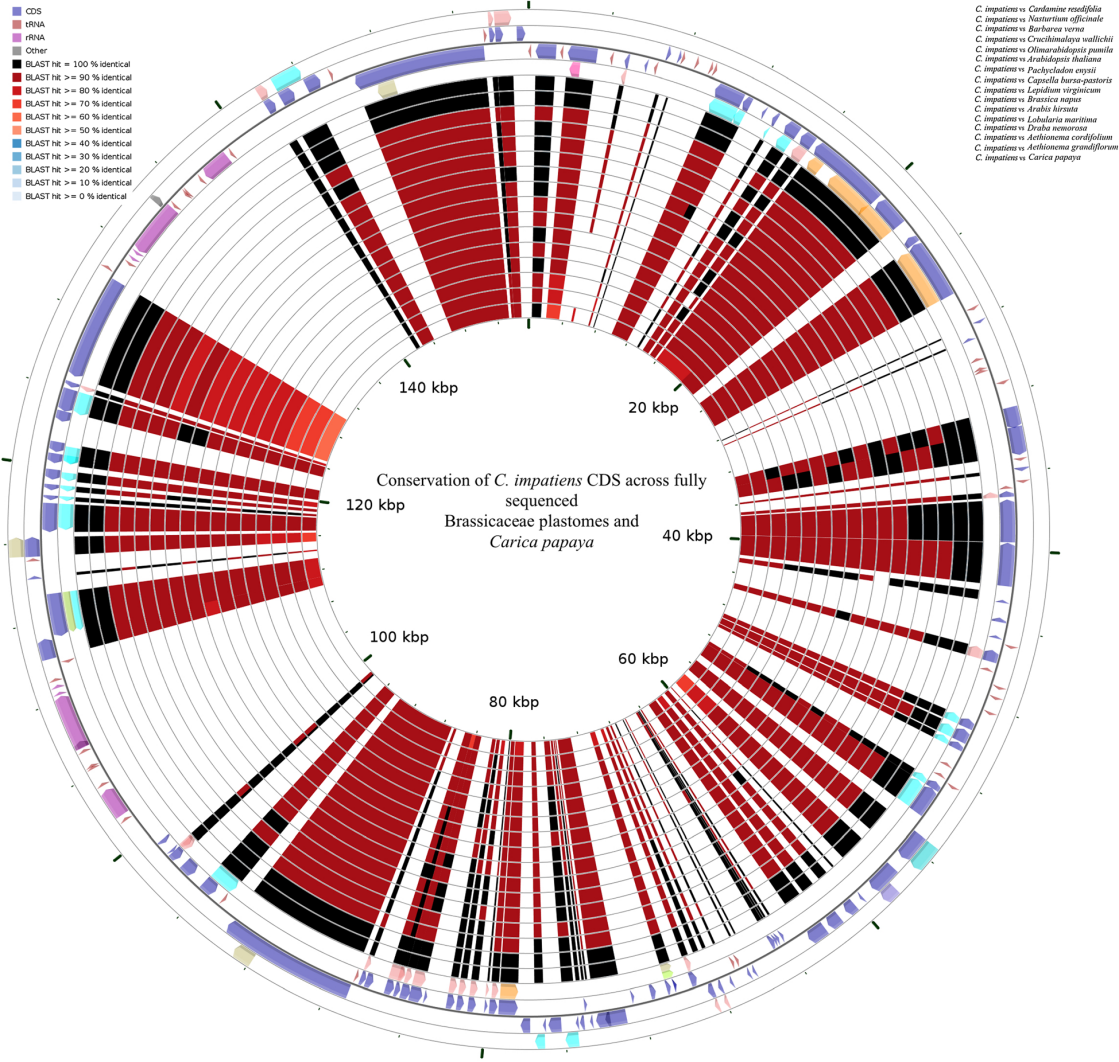
Circular map displaying the conservation of the coding regions across the Brassicacae, the *Cardamine* plastomes sequenced in this study and the outgroup *Carica papaya*.

Among the Brassicaceae species whose plastomes have been fully sequenced so far (a total of 15 at the time of the analyses), only *Nasturtium officinale* and *Barbarea verna* belong to the Cardamineae tribe like *C. impatiens* and *C. resedifolia*. As *Nasturtium* has been indicated as putative sister genus to *Cardamine* [26], the plastome of *N. officinale* was used as reference to calculate average nucleotide identity (ANI) plots using a window size of 1000 bp, step size of 200 bp and a alignment length of 700 bp, 70% identity. As expected by their close relatedness, a high degree of synteny conservation with the reference plastome was observed (Additional file 7: Figure S2). Average nucleotide identity value based on 748 and 568 fragments using one-way and two-way ANI indicated a similarity of 97.76% (SD 2.25%) and 97.55% (SD 2.17%) between *C. resedifolia* and *N. officinale*. Similarly, one-way and two-way ANI values of 98.19% (SD 1.88%) and 98.03% (SD 1.78%) based on 759 fragments and 603 fragments were observed in case of *C. impatiens* and *N. officinale*. Syntenic analysis of the coding regions across Brassicaceae and one outgroup belonging to the Caricaceae family (*Carica papaya*) revealed perfect conservation of gene order along the plastome of the analyzed species (Figure 3). Similarity among plastomes was a function of plastome organization and gene content, with IR and coding regions of fundamental genes being the most highly conserved, as indicated by analysis of pairwise mVISTA plots using *C. impatiens* as reference (Additional file 8: Figure S3).

**Figure 4:**
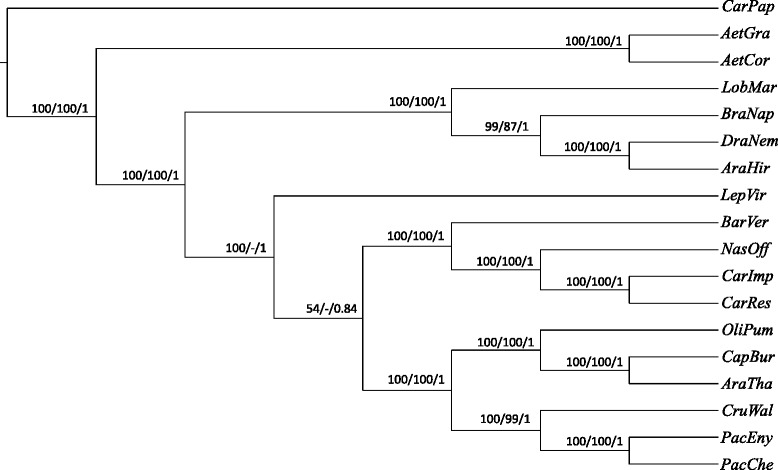
Cladogram of the phylogenetic relationships among Brassicaceae species with fully sequenced plastome used in this study. The cladogram represents the consensus topology of the maximum likelihood (ML), maximum parsimony (MP) and bayesian inference (BI) phylogenetic reconstructions using the concatenated alignment of 75 protein coding genes. Numbers on branches indicate ML/MP/BI support values (bootstrap proportion > 50%). Dashes indicate lack of statistical support. Abbreviation of species names can be found in Additional file 10: Table S7. Phylogenetic tree visualization was done using FigTree.

To precisely determine the phylogenetic position and distance of *C. resedifolia* and *C. impatiens* with respect to the other Brassicaceae with fully sequenced plastome, we performed a concatenated codon-based sequence alignment of the 75 protein coding genes, representing a total of 67698 nucleotide positions. The GTR + I + G model resulted the best fitting model for the matrix according to the JModelTest program using the Akakie information criterion (AIC) and Bayesian information criterion (BIC). Phylogenetic reconstruction was carried out using maximum parsimony (MP), Maximum likelihood (ML) and Bayesian inference (BI). MP analysis resulted in a tree length of 15739, a consistency index of 0.819 and retention index of 0.646. ML analysis revealed a phylogenetic tree with the -lnL of 186099.2 using the GTR + I + G model as estimated using JModelTest. For MP and ML analysis, 1000 bootstrap replicates were evaluated and all the trees obtained were rooted using *Carica papaya* as an outgroup (Figure 4). All phylogenetic methods provided consistent topologies, indicating good reproducibility of the recovered phylogeny. The tree positioning of *Lepidium virginicum*, which lacked resolution in the MP tree, constituted the only exception. As expected, the four taxa from the Cardamineae tribe (genera *Cardamine*, *Nasturtium* and *Barbarea*) formed a well-supported, monophyletic clade with *B. verna* as most basal species. Our phylogenetic reconstruction is in agreement with previous reports on the relationships among Brassicaeacea tribes [47,48], thus indicating that it can be used as a reliable framework for assessment of protein coding gene evolution in the Brassicaeae family in general and *Cardamine* species in particular.

### Molecular evolution of Brassicaceae plastomes

Understanding the patterns of divergence and adaptation among the members of specific phylogenetic clades can offer important clues about the forces driving its evolution [49,50]. To pinpoint whether any genes underwent adaptive evolution in Brassicaceae plastomes in general and in the *Cardamine* genus in particular, we carried out the identification of genes putatively under positive selection using Selecton. At the family level, we observed signatures of positive selection in 10 genes (*ycf1, rbcL, rpoC2, rpl14, matK, petD, ndhF*, *ccsA*, *accD*, and *rpl20*) at a significance level of 0.01 (Table 3). Two of these genes, namely *ycf1* and *accD,* have been reported to undergo fast evolution in other plant lineages as well. *ycf1* is one of the largest plastid genes and it has been classified as the most divergent one in plastomes of tracheophytes [5]. Despite it has been reported to be essential in tobacco [51], it has been lost from various angiosperm groups [52]. Recently, ycf1 was identified as one of the core proteins of the chloroplast inner envelope membrane protein translocon forming a complex (called TIC) with Tic100, Tic56, and Tic20-I [53]. None of the 24 amino acids putatively under positive selection in Brassicaceae are located in predicted transmembrane domains [53], indicating that in Brassicaceae evolution of predicted channel-forming residues is functionally constrained. Analogously to what found for Brassicaceae in our study, in the asterid lineage recent studies also show accelerated rates of evolution in *accD*, a plastid-encoded beta-carboxyl transferase subunit of acetyl-CoA carboxylase (ACCase) [54], which has been functionally re-located to nucleus in the Campanulaceae [55]. As in none of the fully sequenced Brassicaceae re-location of plastidial *accD* to the nuclear genome has been observed, it is likely that the fast evolution of this gene is independent from the genome from which it is expressed. On the other hand, *accD* has been demonstrated to be essential for proper chloroplast and leaf development [54]. Plastidial *accD* together with three nucleus-encoded subunits form the ACCase complex, which been reported to produce the large majority of malonyl CoA required for *de novo* synthesis of fatty acids [56,57] under the regulatory control of the PII protein [58]. Most importantly, there are direct evidences that accD can affect plant fitness and leaf longevity [59]. The signatures of positive selection observed in both Brassicaceae (our study) and asterids [55], therefore, indicate that this gene may have been repeatedly involved in the adaptation to specific ecological niches during the radiation of dicotyledonous plants.

**Table 3:** **Positive selection sites identified with selecton with d.f. =1**

**Gene**	**Null**	**Positive**	Putative sites under positive selection *
*ycf1*	-21668,5	-21647,6	24(343 P, 424 A, 533 D, 565 H, 970 L, 1293 L, 1313 N, 1399 R, 1400 N, 1414 R, 459 W, 564 I, 738 K, 922 F, 928 L, 1081 F, 1113 T, 1235 K, 1259 P, 1343 R, 1428 F, 1475 S, 1477 R, 1533 Y)
*rbcL*	-3000,07	-2984,64	3(326 V, 472 V, 477 A)
*rpoC2*	-11431,8	-11423,5	7(490 F, 527 L, 540 P, 541 H, 981 A, 998 L, 1375 Y)
*rpl14*	-631,147	-623,836	2(18 K, 33 K)
*matK*	-5014,38	-5007,21	1(51 V)
*petD*	-1052,21	-1045,47	2(138 V, 139 V)
*ndhF*	-6497,59	-6491,61	4(65 I, 509 F, 594 Q, 734 M)
*ccsA*	-3031,79	-3026,12	5(97 H, 100 H, 176 L, 182 E, 184 F)
*accD*	-4142,84	-4137,43	3(112 F, 167 H, 485 E)
*rpl20*	-834,791	-831,556	2(80 R, 117 E)

*lower bound > 1.

“Null” and “Positive” columns list likelihood values obtained under the models M8a (null model) and M8 (positive selection), respectively.

Given the prominent role that plastid proteins play in the constitution of cores of photosynthetic complexes [60], one could expect that some photosynthetic genes would also be targeted by positive selection. Previous analyses in leptosporangiates, for instance, uncovered a burst of putatively adaptive changes in the *psb*A gene, which is coding for a core subunit of Photosystem II (PSII). Extensive residue co-evolution along with positive Darwinian selection was also detected [61]. However, we did not observe such burst of high rate of evolution in Brassicaceae *psbA*. We instead observed co-evolving residues along with positive signatures of Darwinian selection in *rbcL* (*ribulose-1, 5-bisphosphate carboxylase/oxygenase*), which codes for RUBISCO, the enzyme catalyzing photosynthetic assimilation of CO_2_ and one of the major rate-limiting steps in this process. Positive rates of selection were observed at three sites across Brassicacae. The observed rates of positive selection on neutral hydrophobic residues such A (alanine) and V (valine) are consistent with previous estimates of selection sites across land plants [62]. As compared to RUBISCO adaptive selection in gymnosperms, where previous reports suggest 7 sites under positive selection (A11V, Q14K, K30Q, S95N, V99A, I133L, and L225I) [63], the low frequency of the sites under positive selection observed in Brassicaceae, which belongs to Angiosperms, could be a consequence of the more recent origin of the latter group. The fact that the long series of geological variations of atmospheric CO_2_ concentrations experienced by gymnosperms seem to parallel adaptive bursts of co-evolution between RUBISCO and RUBISCO activase lend support to this view [63]. Recent studies across Amaranthaceae *sensu lato* identified multiple parallel replacements in both monocotyledonous and dicotyledonous C_4_ species at two residues (281 and 309), suggesting their association with selective advantages in terms of faster and less specific enzymatic activity (e.g. in C_4_ taxa or C_3_ species from cold habitats) [64]. We found no evidence of selection in these or other residues in their proximity in the crystal structure of RUBISCO, indicating that in the Brassicaceae species analyzed (including high altitude *C. resedifolia*) this kind of adaptation possibly did not occur. The three residues under positive selection in our study belong to RUBISCO loop 6 (amino acid 326 V) and C-terminus (amino acids 472 V and 477 A). None of these aminoacids belong to the set of highly conserved residues identified among RUBISCO and RUBISCO-like proteins, which are likely under strong purifying selection [65,66]. This result is in agreement with the observation that in monocotyledons adaptive mutations preferentially affect residues not directly involved in catalysis, but either aminoacids in proximity of the active site or at the interface between RUBISCO subunits [67]. The C-terminus of RUBISCO is involved in interactions between large subunits (intra-dimer) and with RUBISCO activase, and amino acid 472 was previously identified among rbcL residues evolving under positive selection [64]. It is, therefore, possible that the mutation in residues 472 and 477 could contribute to modulate the aggregation and/or activation state of the enzyme in Brassicaceae. Also amino acid 326 has consistently been identified as positively selected in different studies, although in relatively few plant groups [64]. This residue is in close proximity to the fourth among the most often positively selected RUBISCO residues in plants (amino acid 328), which has been associated to adaptive variation of RUBISCO active site possibly by modifying the position of H327, the residue coordinating the P5 phosphate of ribulose-1,5-bisphosphate [64,67]. Such “second shell mutations” in algae and cyanobacteria are known to be able to modulate RUBISCO catalytic parameters [68], and were recently shown to be implicated in the transition from C_3_ to C_4_ photosynthesis in monocotyledons by enhancing conformational flexibility of the open-closed transition [67]. Taken together, these data indicate that in Brassicaceae residue 326 could affect RUBISCO discrimination between CO_2_ and O_2_ fixation, analogously to what suggested for residue 328 in several other plant groups.

The other genes displaying signature of positive selection in our study belong to 4 main functional classes: transcription and transcript processing (*rpoC2*, *matK*), translation (*rps14* and *rpl20*), photosynthetic electron transport and oxidoreduction (*pet*D, *ndh*F), cytochrome biosynthesis (*ccsA*). The broad spectrum of candidate gene functional classes affected indicate that natural selection target different chloroplast functions, supporting the possible involvement of plastid genes in adaptation and speciation processes in the Brassicaceae family [69].

To obtain a more precise picture of the phylogenetic branch(es), where the putatively adaptive changes took place, the rate of substitution mapping on each individual branch was estimated by the MapNH algorithm [70]. Focusing on the Cardamineae tribe and using a branch length threshold to avoid bias towards shorther branches, we found that genes under positive selection in the *Cardamine* lineage (*accD*, *ccsA*, *matK*, *ndhF*, *rpoC2*) evolved faster in *C. resedifolia* as compared to *C. impatiens*, suggesting that adaptive changes may have occurred more frequently in response to the highly selective conditions of high altitude habitats (Additional file 9: Table S6). These results are in line with the accelerated evolutionary rates of cold-related genes observed for *C. resedifolia* in the transcriptome-wide comparison of its transcriptome to that of *C. impatiens* [22]. Given the different genomic inheritance and low number of genes encoded in the chloroplast, it is unfortunately difficult to directly compare the evolutionary patterns observed for photosynthetic plastid genes in this study with the strong purifying selection identified for nuclear-encoded photosynthetic genes of *C. resedifolia* [22]. It is, however, worth of note that the genes with larger differences in evolutionary rates between *C. resedifolia* and *C. impatiens* are not related to photosynthetic light reactions, suggesting that this function is likely under intense purifying selection also for plastidial subunits in *Cardamine* species (Additional file 9: Table S6). Given the relatively few studies available and the complex interplay among the many factors potentially affecting elevational adaptation in plants [71,72], however, additional studies will be needed to specifically address this point.

## Conclusion

In conclusion, the comparative analysis of the *de-novo* sequences of *Cardamine* plastomes obtained in our study identified family-wide molecular signatures of positive selection along with mutationally biased codon usage frequency in Brassicaceae chloroplast genomes. We additionally found evidence that the plastid genes of *C. resedifolia* experienced more intense positive selection than those of the low altitude *C. impatiens*, possibly as a consequence of adaptation to high altitude environments. Taken together, these results provide a series of candidate plastid genes to be functionally tested for elucidating the driving forces underlying adaptation and evolution in this important plant family.

## Methods

### Illumina sequencing, plastome assembly, comparative plastomics and plastome repeats

Genomic DNA was extracted from young leaves of *Cardamine impatiens* and *C. resedifolia* using the DNeasy Plant Mini kit (Qiagen GmbH, Hilden, Germany) and Long PCR amplification with a set of 22 primer pairs was carried out using Advantage 2 polymerase mix (Clontech Laboratories Inc., Mountain View, CA, USA) according to manufacturer’s instructions. We chose to use a long-PCR whole plastome amplification approach to maximize the number of reads to be used for assembly. The primer pairs used are listed in Additional file 1: Table S1. Amplicons from each species were pooled in equimolar ratio, sheared with Covaris S220 (Covaris Inc., Woburn, MA, USA) to the average size of 400 bp and used for illumina sequencing library preparation. Each library was constructed with TruSeq DNA sample preparation kits V2 for paired-end sequencing (Illumina Inc., San Diego, CA) and sequenced on a HiSeq 2000 at The Genome Analysis Centre (Norwich, UK). Subsequently, the reads were quality filtered using a Q30 quality value cutoff using FASTX_Toolkit available from http://hannonlab.cshl.edu/fastx_toolkit/. After subsequent quality mapping on the Brassicaceae plastomes, contaminating reads were filtered off. Specifically, raw reads were mapped on the publicly available Brassicaceae plastomes (Additional file 10: Table S7) using the Burrows-Wheeler Aligner (BWA) programusing -n 2, -k 5 and -t 10. SAM and BAM files obtained as a result were consecutively filtered for the properly paired end (PE) reads using SAMtools [73].

To obtain the *de novo* plastome assembly, properly PE reads were assembled using Velvet assembler [74]. In Velvet, N50 and coverage were evaluated for all *K-mers* ranging from 37 to 73 in increments of 4. Finally, the plastome assembly with *K-mer* = 65 was used for all subsequent analyses in both species. The selected Velvet assembly was further scaffolded using optical read mapping as implemented in Opera [75]. Assembled scaffolds were further error corrected using the SEQUEL software by re-mapping the reads and extending/correcting the ends of the scaffolded regions [76]. Gap filling was performed using the GapFiller program with parameters –m 80 and 10 rounds of iterative gap filling [77]. All the given computational analysis was performed on a server equipped with 128 cores and a total of 512 GB.

Following scaffolding and gap filling, *C. resedifolia* and *C. impatiens* scaffolds were systematically contiguated based on the *Nasturtium officinale* plastome (AP009376.1, 155,105 bp) using the nucmer and show-tiling programs of the MUMmer package [78]. Finally, mummer plot from the same package was used to evaluate the syntenic plots and the organization of the inverted repeats by pairwise comparison between the *N. officinale* and *C. resedifolia* and *C. impatiens* plastomes. Due to assembler’s insufficient accuracy in assembly of repeat regions, manual curations of the IRs were carried out using the BLAST2Seq program by comparison of the scaffolded regions with the *N. officinale* plastome. To test assembly quality and coverage, average nucleotide identity plots were calculated. Additionally, the junctions of the IRs and all remaining regions containing Ns were amplified by PCR using the primers listed in Additional file 1: Table S1 and Sanger sequenced. The finished *C. resedifolia* and *C. impatiens* chloroplast sequences have been deposited to GenBank with accession numbers KJ136822 and KJ136821, respectively.

To assess the levels of plastid syntenic conservation, the assembled plastomes of *C. resedifolia* and *C. impatiens* were compared to all publicly available plastomes of Brassicaceae using CGview by computing pairwise similarity [79]. Additionally, mVISTA plots were constructed using the annotated features of *C. resedifolia* and *C. impatiens* plastomes with a rank probability of 0.7 (70% alignment conservation) to estimate genome-wide conservation profiles [80]. To identify the stretches of the repetitive units, the REPUTER program was used with parameters -f –p –r –c –l 30 –h 3 –s and the repeat patterns along with the corresponding genomic co-ordinates were tabulated [81]. Additionally, we mined the distribution of perfect and compound simple sequence repeats using MISA (http://pgrc.ipk-gatersleben.de/misa/). In our analysis, we defined a minimum repetitive stretch of 10 nucleotides as mono-nucleotide, a consecutive stretch of 4 repeats units to be classified as di- and tri-nucleotide, and a stretch of 3 repeat units for each tetra-, penta- and hexa-nucleotide stretches as simple sequence repeats (SSRs).

### Chloroplast genome annotation and codon usage estimation

The assembled plastome of *C. resedifolia* and *C. impatiens* was annotated using cpGAVAS [82] and DOGMA (Dual Organellar GenoMe Annotator) [83]. Manual curation of start and stop codons was carried out using the 20 available reference Brassicaceae plastomes. The predicted coding regions were manually inspected and were re-sequenced with Sanger chemistry whenever large differences in conceptually translated protein sequences were detected compared with the reference plastome of *N. officiale* (Additional file 10: Table S7). GenomeVx [84] was used for visualization of plastome maps. Transfer-RNAs (t-RNAs) were identified using the t-RNAscan-SE software using the plastid genetic code and the covariance models of RNA secondary structure as implemented in cove algorithm [85]. Only coding regions longer than 300 bp from *Cardamine* and the other Brassicaceae plastomes were used for estimation of codon usage in CodonW with translational table = 11 (available from codonw.sourceforge.net). We further tabulated additional codon usage measures such as Nc (effective number of codons), GC_3s_ (frequency of the GC at third synonymous position). GC, GC_1_, GC_2_ and GC_3_ were calculated with in-house Perl scripts. Estimation of the standard effective number of codon (Nc) was tabulated using the equation N(c) = 2 + s + 29/(s(2) + (1-s)(2)), where s denotes GC_3s_ [86].

### Molecular evolution in *Cardamine* plastomes

For evaluating the patterns of molecular evolution, codon alignment of the coding regions was created using MACSE, which allows the identification of frameshift events [87]. Model selection was performed using the JmodelTest 2 [88]. Phylogenetic reconstruction was performed using PhyML with 1000 bootstrap replicates [89]. To identify the role of selection on the evolution of plastid genes, MACSE codon alignments were analysed using Selecton [90] allowing for two models: M8 (model of positive selection) and M8a (null model) and likelihood scores were compared for each gene set followed by a chi-square test with 1 degree of freedom. Only tests with probability lower than 0.01 were considered significant and were classified as genes under positive selection. We further mapped the substitution rate on the phylogeny of the Brassicaceae species using MapNH [70] with a threshold of 10 to provide a reliable estimation of the braches under selection.

### Availability of supporting data

The data set supporting the results of this article are available in the GenBank repository, *Cardamine resedifolia* plastome (GenBank accession number KJ136821) and *C. impatiens* (accession number KJ136822). The phylogenetic matrix and trees are available from Treebase (http://purl.org/phylo/treebase/phylows/study/TB2:S17255).

## Additional files

Additional file 1: Table S1. Long-range PCR primers used for tiled whole-plastome amplification. Additional file 2: Table S2. Summary of distribution and localization of genes in the *C. resedifolia* and *C. impatiens* plastomes. Additional file 3: Table S3. Genes with introns in *C. resedifolia* (^a^) and *C. impatiens* (^b^) plastome and length of exons and introns. Additional file 4: Figure S1. Nc plot showing the distribution of the genes >300 bp in *C. resedifolia* and *C. impatiens*. The black line in the curve represents the standard effective number of codons (Nc) calculated using the equation N(c) = 2 + s + 29/(s(2) + (1-s)(2)), where s denotes GC_3s_ (Wright [86]). Additional file 5: Table S4. Distribution and localization of repeat sequences in cpDNA of *C. impatiens and C. resedifolia*. Additional file 6: Table S5. Cumulative SSR frequency and corresponding primer pairs in *C. resedifolia* and *C. impatiens*. Additional file 7: Figure S2. Average nucleotide identity plots of the *C. resedifolia* and *C. impatiens* against *Nasturtium officinale*. Additional file 8: Figure S3. mVISTA plots showing genome-wise similarity between *C. resedifolia*, *C. impatiens* and *N. officinale* with rank probability of 70% and window size of 100 bp. The annotations displayed are derived from the *C. impatiens* plastome. Additional file 9: Table S6. Phylogenetic distribution map of substitution rates using probabilistic substitution mapping under the homogenous model of sequence evolution. Additional file 10: Table S7. Accessions and references for fully sequenced plastomes used in phylogenetic reconstruction and genome comparison in this study.

## Abbreviations

RUBISCO: Ribulose-1, 5-bisphosphate carboxylase/oxygenase
IR: Inverted repeat region
LSC: Large single copy region
SSC: Small single copy region
Bp: Base pair
Nc: Effective number of codons used in a gene
GC: Guanine-cytosine
SSR: Simple sequence repeat
ANI: Average nucleotide identity
