# Supplementary material for: Plastome organization and evolution of chloroplast genes in Cardamine species adapted to contrasting habitats
Source: BMC Genomics. 2015 Apr 17;16(1):306. doi: 10.1186/s12864-015-1498-0 (PMC4446112; doi:10.1186/s12864-015-1498-0)
Supplement: Additional file 3: Table S3. — Genes with introns in C. resedifolia (a) and C. impatiens (b) plastome and length of exons and introns. [file 12864_2015_1498_MOESM3_ESM.docx]

Table S3: Genes with introns in *C. resedifolia* (^a^) and *C. impatiens* (^b^) plastome and length of exons and introns.

| **Gene** | **Location** | **Exon I (bp)** | **Intron I (bp)** | **Exon II (bp)** | **Intron II (bp)** | **Exon III (bp)** |
| --- | --- | --- | --- | --- | --- | --- |
| *atpF* | LSC | 410^a^/410^b^ | 679^a^/714^b^ | 145^a^/145^b^ |  |  |
| *clpP* | LSC | 228^a^/228^b^ | 576a/573b | 292^a^/292^b^ | 898^a^/897^b^ | 71^a^/71^b^ |
| *ndhA* | SSC | 530^a^/530^b^ | 1063a/1072b | 553^a^/553^b^ |  |  |
| *ndhB* | IR | 762^a^/762^b^ | 685a/685b | 723^a^/723^b^ |  |  |
| *petB* | LSC | 6^a^/6^b^ | 794a/794b | 642^a^/642^b^ |  |  |
| *petD* | LSC | 8^a^/8^b^ | 728a/710b | 475^a^/475^b^ |  |  |
| *rpl16* | LSC | 399^a^/399^b^ | 1090a/1110b | 9^a^/9^b^ |  |  |
| *rpl2* | IR | 435^a^/435^b^ | 682a/682b | 390^a^/390^b^ |  |  |
| *rpoC1* | LSC | 1611^a^/1611^b^ | 800a/794b | 432^a^/432^b^ |  |  |
| *rps12** | LSC | 114^a^/114^b^ | -/- | 26^a^/26^b^ | 537^a^/536^b^ | 232^a^/232^b^ |
| *rps16* | LSC | 227^a^/227^b^ | 872a/883b | 40^a^/40^b^ |  |  |
| trnA-UGC | IR | 38^a^/38^b^ | 800a/800b | 35^a^/35^b^ |  |  |
| trnG-UCC | LSC | 23^a^/23^b^ | 716a/716b | 49^a^/49^b^ |  |  |
| trnI-GAU | IR | 42^a^/42^b^ | 941a/941b | 35^a^/35^b^ |  |  |
| trnK-UUU | LSC | 35^a^/35^b^ | 2552a/2561b | 37^a^/37^b^ |  |  |
| trnL-UAA | LSC | 35^a^/35^b^ | 514a/499b | 50^a^/50^b^ |  |  |
| trnV-UAC | LSC | 35^a^/35^b^ | 606a/604b | 39^a^/39^b^ |  |  |
| *ycf3* | LSC | 153^a^/153^b^ | 789a/782b | 228^a^/228^b^ | 703^a^/721^b^ | 126^a^/126^b^ |

**rps12* is a trans-spliced gene with the 5' end located in the LSC region and the duplicated 3' end in the IR regions
