# Supplementary figures and images for: Plastome organization and evolution of chloroplast genes in Cardamine species adapted to contrasting habitats
Source: BMC Genomics. 2015 Apr 17;16(1):306. doi: 10.1186/s12864-015-1498-0 (PMC4446112; doi:10.1186/s12864-015-1498-0)

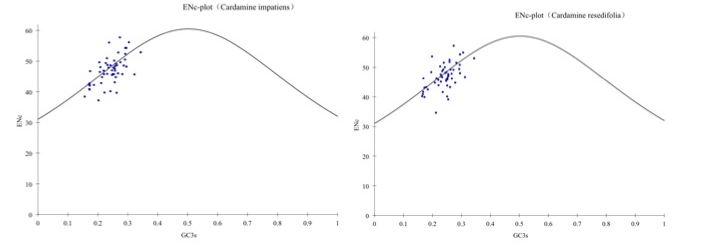

Supplement: Additional file 4: Figure S1. — Nc plot showing the distribution of the genes >300 bp in C. resedifolia and C. impatiens. The black line in the curve represents the standard effective number of codons (Nc) calculated using the equation N(c) = 2 + s + 29/(s(2) + (1-s)(2)), where s denotes GC3s (Wright [86]). [file 12864_2015_1498_MOESM4_ESM.png]

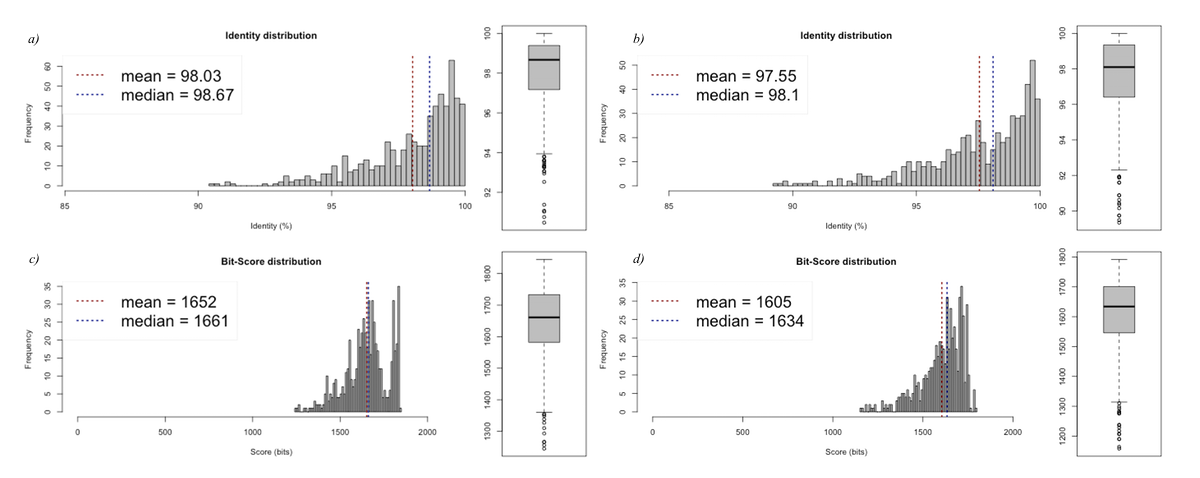

Supplement: Additional file 7: Figure S2. — Average nucleotide identity plots of the C. resedifolia and C. impatiens against Nasturtium officinale. [file 12864_2015_1498_MOESM7_ESM.png]

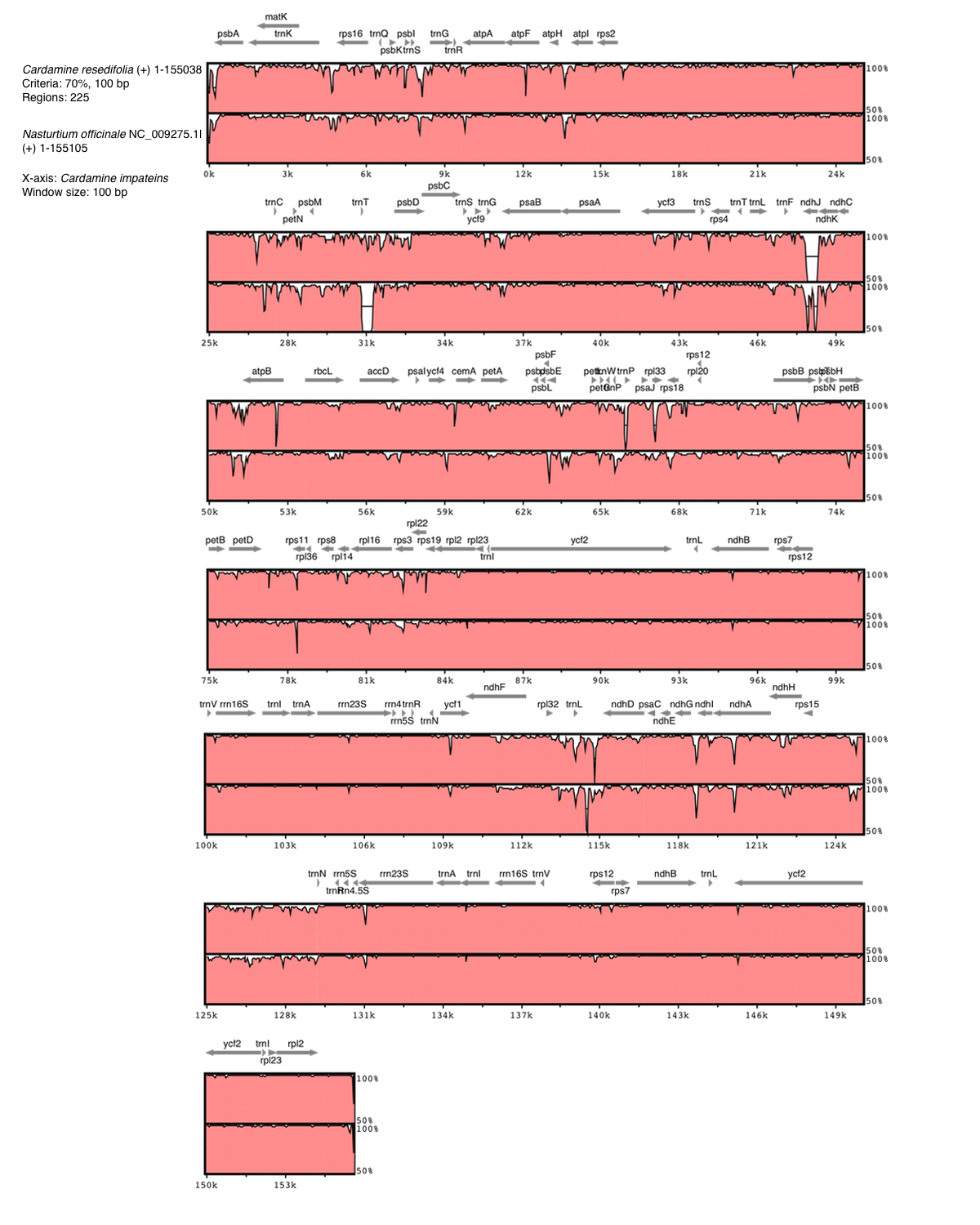

Supplement: Additional file 8: Figure S3. — mVISTA plots showing genome-wise similarity between C. resedifolia, C. impatiens and N. officinale with rank probability of 70% and window size of 100 bp. The annotations displayed are derived from the C. impatiens plastome. [file 12864_2015_1498_MOESM8_ESM.png]
